# When Should I Have a Mammogram? Recent Changes in ACS Mammography Guidelines: Implications for Practice

**Published:** 2016-07-01

**Authors:** Karen Meneses

**Affiliations:** The University of Alabama at Birmingham

In the United States, breast cancer is the most common female cancer and the second leading cause of female cancer death (American Cancer Society [ACS], 2015). Breast cancer significantly contributes to morbidity and premature mortality among American women (ACS, 2015). Early detection with mammography has been known to reduce morbidity and mortality. Yet, today, the answer to the routine question asked by millions of American women: "When should I have a mammogram?" requires a more complex answer. And the answer is "That depends."

## 2015 UPDATES

In 2015, the ACS issued an update to its 2003 screening mammography guidelines (Oeffinger et al., 2015). The 2003 mammography screening recommendation for women at average risk was to start annual mammography at age 40 years and continue annual screening as long as a woman was in good health and, if diagnosed, could tolerate breast cancer treatment. The 2003 ACS guidelines also recommended that for women in their 20s and 30s, clinical breast examination (CBE) should be part of health evaluation at least every 3 years and for asymptomatic women 40 years and older, CBE should be performed annually.

The newly issued 2015 ACS screening mammography guidelines depart from the 2003 guidelines in several ways (Smith et al., 2003). First, annual screening recommendations differ based on age. The ACS now recommends that annual screening should begin at age 45 and continue until age 54. At age 55, women should transition to biennial screening and can continue as long as their overall health is good and they have a life expectancy of 10 or more years. Clinical breast examination is no longer recommended for every woman. Table 1 compares the ACS 2003 and 2015 guidelines.

**Table 1:**
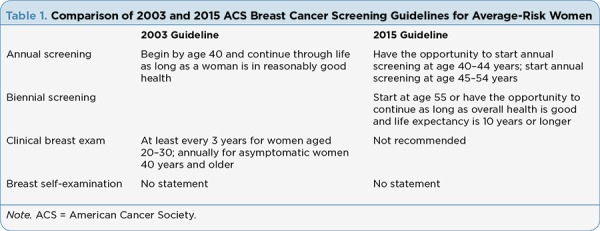
Comparison of 2003 and 2015 ACS Breast Cancer Screening Guidelines for Average-Risk Women

So what prompted this substantial change in recommended screening guidelines? An editorial by Keating and Pace (2015) identified three key areas. First, the ACS commissioned a panel to review the current evidence of the benefits of mammography. The review panel included recent observational studies showing a wider range of estimates—about 20% to 40%—for breast cancer mortality relative risk reductions compared with prior reviews of the evidence using randomized clinical trials showing risk reduction of about 15%. The panel further acknowledged the limitations in both the observational designs (i.e., noncomparable control groups, lead time, and length of time bias) and the randomized trial designs (i.e., heterogeneous, older, and increasingly outdated).

Second, the panel used the Surveillance, Epidemiology, and End Results (SEER) data using 5-year age groups to estimate the benefits and harms of mammography screening compared with previous estimates using 10-year age groups. The panel found that the breast cancer deaths between women aged 45 to 49 years compared with those of women aged 50 to 54 years, rather than women aged 40 to 44 years. Thus, the panel recommended screening mammography to begin at age 50.

Third, the panel commissioned an analysis of annual vs. biennial screening by the Breast Cancer Surveillance Consortium (BCSC; Miglioretti et al., 2015). The BCSC evaluated 15,440 women aged 40 to 85 diagnosed with breast cancer between 1996 and 2012. Women received either annual or biennial screening mammography. Findings showed that premenopausal women with biennial screening had higher stage IIIB disease compared with women having annual screening. No differences were found between premenopausal and postmenopausal women. Thus, the panel recommended the change in the ACS guideline that women between 45 and 54 years should receive annual screening.

The panel found that the evidence of CBE and mortality reduction with CBE to be of low quality and unconvincing. Thus, the panel recommended against CBE.

## AVERAGE VS. HIGH RISK

Overall, the changes in the ACS recommendations refer to women of average breast cancer risk. So, who are they? Average-risk women are those who have none of the following characteristics: (1) a personal history of breast cancer; (2) a suspected or confirmed genetic mutation such as *BRCA*; and (3) a personal history of radiation therapy to the chest wall received at a young age. The recommendations do not refer to women who are considered at higher risk.

Who is considered higher risk among women younger than 45 years? They are women with (1) a personal history of breast cancer; (2) a suspected or confirmed genetic mutation such as *BRCA*; (3) personal history of radiation therapy to the chest wall received at a young age; (4) first-degree relatives diagnosed with breast cancer before the age of 45 or ovarian cancer at any age (particularly if more than one relative was diagnosed) or a male relative with breast cancer; (5) Ashkenazi Jewish heritage; and/or (6) other breast health problems or conditions, such as breast density, lobular carcinoma in situ, ductal carcinoma in situ, atypical ductal hyperplasia, or atypical lobular hyperplasia.

## COMPARING SCREENING GUIDELINES

How do the 2015 ACS mammography screening guidelines compare with the 2009 US Preventive Services Task Force (USPSTF) recommendations? Table 2 compares the two guidelines related to annual and biennial screening, CBE, and breast self-exam. First, the USPSTF recommends biennial screening mammography for women between the ages of 50 and 74. Deciding to start regular, biennial screening mammography before the age of 50 should be done individually taking context into account, including patients’ values about benefits and harms.

**Table 2:**
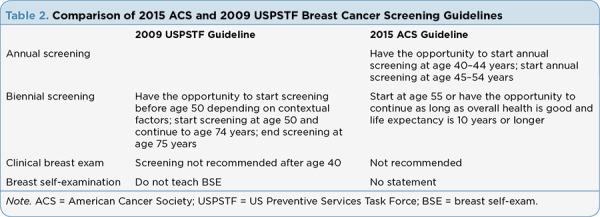
Comparison of 2015 ACS and 2009 USPSTF Breast Cancer Screening Guidelines

Second, the USPSTF does not recommend screening mammography in women 75 and older. Third, the USPSTF found insufficient evidence to recommend CBE in women over 40. Fourth, the USPSTF recommends against teaching breast self-exam. And finally, current evidence regarding digital mammography or magnetic resonance imaging is insufficient to assess the additional benefits and harms over film mammography.

## IMPLICATIONS FOR ADVANCED PRACTITIONERS

So we return to the question, "When should I have a mammogram?" The answer can still be "that depends," because there is no specific right or wrong answer for women of average risk. Although some advanced practitioners may argue that the recommendations are confusing, it is hoped that others may view the new 2015 ACS recommendations in another light: one where the dialogue becomes patient-driven, patient-centered, and patient-valued.

Advanced practitioners can begin the conversation with patient-driven comments: "Let’s talk about average risk. Are you at average risk? That depends on your current age as well as your family and personal histories. Maybe you are not at average risk. Thus, the guidelines do not pertain to you." It may be likely that advanced practitioners in oncology may see women who are not at average risk. They may be seeing survivors and family members of cancer survivors.

Among our advanced practitioner colleagues in primary care or women’s health practices, they may be using the USPSTF guidelines for average-risk women. In this instance, the USPSTF guidelines may be more in line with the updated ACS guidelines, where average-risk women can have the opportunity to start biennial screening before the age of 50 years. The USPSTF guidelines are silent on the topic of annual screening before the age of 50, but the 2015 ACS guidelines for average-risk women begin at age 45. Thus, it is most likely that the topic of screening average-risk young women is covered in the ACS guidelines. So, both the new ACS and the USPSTF guidelines for young women cover the answer to "When should I have a mammogram?" Most importantly, maintaining a balanced discussion focused on risk and benefits as well as personal preferences and values can keep the dialogue moving.

Given the weight of the new evidence about average risk, there is greater opportunity to apply evidence to practice to help women make informed decisions that are best for them. So, let the conversation begin!
